# The Autoimmune Rheumatic Disease Related Dry Eye and Its Association with Retinopathy

**DOI:** 10.3390/biom13050724

**Published:** 2023-04-23

**Authors:** Huimin Shan, Wenhui Liu, Yangyang Li, Kunpeng Pang

**Affiliations:** Department of Ophthalmology, Qilu Hospital of Shandong University, Jinan 250012, China

**Keywords:** autoimmune rheumatic diseases, dry eye disease, Sjögren’s syndrome, rheumatoid arthritis, systemic lupus erythematosus, systemic sclerosis, retinal disease

## Abstract

Dry eye disease is a chronic disease of the ocular surface characterized by abnormal tear film composition, tear film instability, and ocular surface inflammation, affecting 5% to 50% of the population worldwide. Autoimmune rheumatic diseases (ARDs) are systemic disorders with multi-organ involvement, including the eye, and play a significant role in dry eye. To date, most studies have focused on Sjögren’s syndrome (one of the ARDs) since it manifests as two of the most common symptoms–dry eyes and a dry mouth-and attracts physicians to explore the relationship between dry eye and ARDs. Many patients complained of dry eye related symptoms before they were diagnosed with ARDs, and ocular surface malaise is a sensitive indicator of the severity of ARDs. In addition, ARD related dry eye is also associated with some retinal diseases directly or indirectly, which are described in this review. This review also summarizes the incidence, epidemiological characteristics, pathogenesis, and accompanying ocular lesions of ARD’s related dry eye, emphasizing the potential role of dry eye in recognition and monitoring among ARDs patients.

## 1. Introduction

Dry eye is a multifactorial disease of the ocular surface characterized by a loss of homeostasis of the tear film and accompanied by ocular symptoms, in which tear film instability and hyperosmolarity, ocular surface inflammation and damage, and neurosensory abnormalities play etiological roles [1]. It can be manifested as decreased vision and eye discomfort, accompanied by eye inflammation and ocular nerve damage. The vision-related quality of life and work efficiency of patients are significantly reduced by those malaises [2]. Epidemiological surveys reported that the prevalence of dry eye differed greatly among regions, ranging from 5% to 50% worldwide [3]. The incidence of dry eye has increased significantly in recent years due to the prolonged use of digital screens [4], air pollution [5], and population aging. Predisposing factors of dry eye include the environment, increased use of electronic products, systemic diseases, ocular surface diseases, eye surgeries, application of eye drops and ointment, etc. Systemic diseases, especially rheumatic immune diseases are closely related to dry eye [2].

Autoimmune rheumatic diseases (ARDs), also named rheumatic immune diseases or immune-mediated rheumatic diseases, are a group of diseases related to the overactivation of the immune system, including primary Sjögren’s syndrome (SS), rheumatoid arthritis (RA), systemic lupus erythematosus (SLE), systemic sclerosis (SSc) or scleroderma, idiopathic inflammatory myositis (IIM), and the systemic vasculitides (SV) [6].

As a systemic disorder with multi-organ involvement, ARDs lead to various clinical manifestations including skin lesions, joint pain, vascular damage, and dry eye. Furthermore, treatments for ARDs, including methotrexate and cyclophosphamide, usually cause or exacerbate symptoms of dry eye. Under normal conditions, the intrinsic immune mechanisms of the eyes could maintain the microenvironment homeostasis. When the eye becomes the target of the immune response of ARDs, the ocular immune system is excessively stimulated by relevant immunoregulatory molecules and the balance of the immunoregulatory mechanisms is therefore disrupted. Additionally, chronic ocular surface inflammation is a result of dysregulated balance of the innate and adaptive immune systems, leading to chronic dry eye [7]. Ocular surface symptoms could be sensitive manifestations of the severity of the systemic condition of ARDs. In addition, a considerable proportion of dry eye patients have ARDs but are not diagnosed and treated in a timely manner [8]. However, the reported literature mainly focused on specific kinds of ARDs (SS, RA, or SLE), and the number of cases is limited. Moreover, we retrieved a few studies exploring the correlation between other or overall ARDs and dry eye. Hence, this review aims to summarize the characteristics and pathogenesis of dry eye in various kinds of ARDs. For the lack of studies reporting IIM or SV-related dry eye, this article mainly focuses on the relationship between other kinds of ARDs (SS, RA, SLE, and SSc) and dry eye. Dry eye is related to some retinal diseases directly or indirectly. The retina is often involved in ARDs, commonly associated with autoimmunity and systemic inflammatory cell infiltration. Consequently. the retinal diseases related to dry eye and ARDs are also illuminated.

## 2. Epidemiology of ARD Related Dry Eye

It is reported that ARDs are significant causes of dry eye. Approximately 10–95% of patients with immune system disorders are accompanied by dry eye. For example, 38–47% of patients with RA have dry eye [9,10], 13.4–39.5% in SLE [11,12], 95% in SS [13,14], and 37–79% in SSc [15]. Consequently, the prevalence of dry eye in patients with SS is higher than those in other diseases of ARDs. In addition, the proportion of females with ARDs is much higher than that of males (appropriately 9:1) [16]. Hence, ARD related dry eye is more likely to affect female patients.

The relationship between SS and dry eye was emphasized in the guidelines reported by the American Academy of Ophthalmology. The guidelines showed that about 10% of patients with dry eye have SS [8]. According to a randomized controlled study that included 535 patients with moderate to severe dry eye disease, SS was significantly associated with more severe dry eye signs [17]. Moreover, the guidelines also report that RA is a risk factor for dry eye [8]. Similarly, Paulsen et.al suggested that arthritis significantly correlated with dry eye symptoms [18]. A recent prospective multicenter study involving more than 400 patients with primary SS showed that 94% of cases had dry eye symptoms [19]. Abd-Allah et.al. found that RA can be accompanied by severe dry eye symptoms even when it is not accompanied by secondary SS [20]. Similarly, the incidence of dry eye in SLE patients was much higher than that in healthy individuals [21].

## 3. Mechanisms of ARD Related Dry Eye

### 3.1. Pathogenesis of Dry Eye

**Figure 1:**
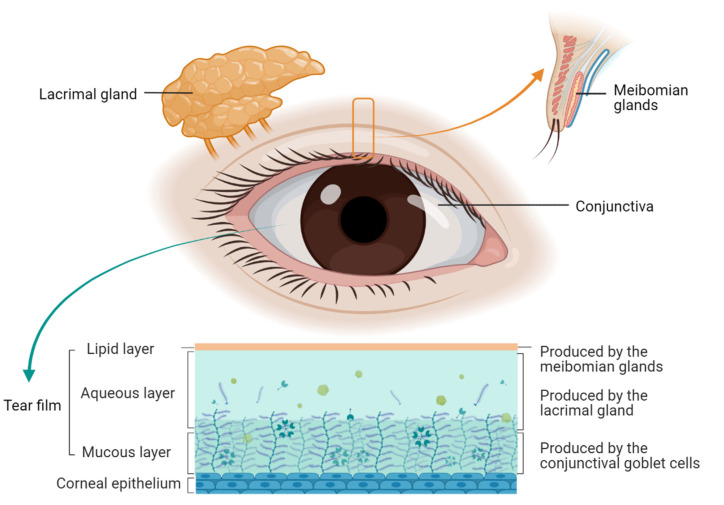
Ocular structures related to dry eye. The three main components of the tear film are the lipid layer, the aqueous layer, and the mucous layer, which are produced by the meibomian glands, the lacrimal gland, and the conjunctival goblet cells, respectively. Decreased tear production by the lacrimal gland results in less eye surface lubrication. Additionally, decreased oil secretion by meibomian glands leads to excess tear evaporation.

The pathogenesis of dry eye is complex and involves a group of units, such as the lacrimal gland, meibomian gland, tear film, conjunctival membrane, cornea, and tissue structures [22]. Pathological changes include inflammatory factors, tear hyperosmotic pressure, lipids, mucins, microorganisms, etc. Dry eye is commonly classified into aqueous-deficient dry eye and evaporative dry eye [2]. The main mechanisms are evaporation and water loss, leading to hyperosmotic tissue damage. Ocular surface inflammation is recognized as the critical process of dry eye pathogenesis. Induced by a variety of risk factors such as hyperosmolarity, UV light, and desiccation, ocular surface epithelial cells release various mediators, including metalloproteinases, inflammatory cytokines, and chemokines. These lead to relevant immune-cell activation and associated inflammation through stress signal transduction pathways [22]. Ocular structures related to dry eye are presented in Figure 1.

Both innate and adaptive immunity are involved in the pathogenesis of dry eye. Previous studies based on clinical and laboratory research have found that Th1 and Th17 cells could modulate the immune response on the ocular surface [23]. Combined with exposure to autoantigens, these innate inflammatory mediators can lead to an adaptive T cell-mediated response. Additionally, after the activation of the immune response, the dysfunction and death of conjunctival goblet cells is promoted by the Th1 cytokine IFN-γ, which aggravates dry eye [24]. Studies on human and animal models have shown that loss of ocular surface epithelial cells and goblet cells could lead to shorter tear break-up time [25]. Interferon-gamma (IFN-γ), produced by NK cells, is responsible for upregulating Th1 cells to recruit chemokines (CXCL9, CXCL10, and CXCL11) in conjunctival and corneal epithelial cells. Meanwhile, increasing expression of activating NK cell receptor ligands and activation of innate immune pathways can lead to enhancement of T cell infiltration, involving the lysis of epithelial tight junctions. In consequence, the cornea barrier is destroyed and the phenotype of the epithelial cells changes, which destabilizes the tear film, amplifies inflammation and creates a vicious cycle [22].

### 3.2. Pathogenesis of ARD Related Dry Eye

The immune systems in patients with rheumatic immune diseases are impaired, resulting in abnormal immune responses and imbalanced immune regulation. Substantial immune cells and inflammatory factors imperil the lacrimal gland, conjunctival membrane, cornea, and meibomian glands, resulting in tissue destruction and dysfunction [26]. A large number of immune cells, most of which are T lymphocytes, infiltrate the lacrimal duct and accessory lacrimal gland in patients with rheumatic and immune diseases, causing autophagy and apoptosis of acinar, ductal and myoepithelial cells, disruption of lacrimal gland function and a decrease in tear secretion [25].

Due to the presence of innate immune cells and the secretion of inflammatory response factors, the signal transduction caused by abnormal antibodies leads to chronic fibrosis of the lacrimal gland and further aggravation of dysfunction. The infiltration of T cells in the conjunctival tissue results in squamous metaplasia, decreased goblet cell density, and reduced mucin secretion. The deposition of autoantibodies and antigen-antibody complexes in corneal tissues leads to corneal dystrophy [27]. An increased number of corneal dendritic cells, as well as decreased nerve density and sensitivity are revealed by confocal microscopy through focusing [28]. Villani et.al. found that 35 patients with SS-related dry eye had significantly low corneal nerve counts (3.34 ± 0.76 vs. 5.10 ± 0.79, *p* < 0.0001) and an increased degree of tortuosity (2.62 ± 0.94 vs. 1.20 ± 0.70, *p* < 0.0001), compared with the control group [29], which was consistent with the study by Benítez del Castillo et al. [30]. Moreover, Xu et al. [31] reported that the corneal nerve sensitivity of SS-related dry eye patients was significantly reduced (4.5 ± 1.2 cm vs. 5.8 ± 0.4 cm, *p* < 0.01). Additionally, immune cell infiltration in the meibomian glands and consequent mechanical obstruction of its ducts were very common in patients with rheumatic immune disease related dry eye (57.9%) [32]. Atrophy and occlusion of some meibomian gland ducts can be revealed by meibomian gland imaging. Moreover, recent studies have shown that gut dysbiosis (Firmicutes/Bacteroidetes ratio and genus Faecalibacterium) is closely related to dry eye diseases caused by autoimmune diseases such as SS. However, the key communication routes of the “gut dysbiosis–ocular surface–lacrimal gland axis” still need to be further elucidated [33].

In terms of genetic heredity, RA is a complex disease and is correlated with multiple genetic loci, each of which usually has only one modest association with a specific condition [34]. According to a recent study, 39 variants were identified in immune-related genes across SLE, RA, and SS families. Among this gene set, regulation of T cell activation and T cell receptor signaling pathways were particularly concentrated [35], which might correlate with symptoms and signs of dry eye. Consequently, gene analysis is a powerful tool for understanding the pathogenesis of ARD related dry eye [36].

#### 3.2.1. Primary Sjogren’s Syndrome & Dry Eye

Primary SS is an autoimmune and systemic disorder. Globally, the prevalence of primary SS ranges from 0.1% to 4.8% depending on age [37,38], and 90% of patients are females. Sicca symptoms (dry mouth and dry eyes) are common oral and ocular complaints. Nevertheless, SS is often underdiagnosed, misdiagnosed, or delayed in being diagnosed since the symptoms are usually vague for many years [39] until irreversible organ damage has occurred [40]. As an autoimmune disease that mainly affects exocrine glands (such as the salivary and lacrimal glands), primary SS is most closely related to dry eye among the various types of ARDs. Primary SS is also a possible diagnosis in patients with dry eye symptoms. Staining of the temporal conjunctiva and severe dry mouth symptoms were the major factors in distinguishing primary SS from dry eye disease [41].

SS is a chronic inflammatory autoimmune disease, histopathologically characterized by lymphocytic infiltration of exocrine (mainly salivary and lacrimal) glands with remarkable B-cell hyperactivity and the presence of autoantibodies against the ribonucleoprotein particles SSA/Ro and SSB/La. The glandular function is impaired by the infiltrating cells in several ways, including local production of autoantibodies, destruction of glandular structures by cell-mediated mechanisms, and secretion of cytokines that activate pathways related to interferons (IFNs) [13]. The salivary and lacrimal glands are the principal targets of a proposed T cell-mediated chronic inflammation, followed by glandular atrophy and deficient function. Primary SS is considered to arise from an interplay between environmental factors and genetic susceptibility. Activation of B cells, the presence of anti-Ro-52 antibody and the “triggers” of viral infections result in the activation of the innate and adaptive immune systems, which perpetuate self-reactive activity against healthy tissues [42,43].

Due to the invasive process of lacrimal gland biopsy, most studies of SS related dry eye were carried out on mouse models, such as the Aec and IL-12 transgenic mice [44], TSP-1KO mice [45], CD25KO mice [46], BXSB/MpJ-Yaa mice [47], and lupus-prone female NZB×NZWF1 (B/WF1) mice [48] (secondary SS), and indicated lymphocytic infiltrations as well as loss of secretory functions. Noteworthily, Hayashi et al. [48] reported substantial destruction of the myoepithelial cells and lysis of basement membranes within the inflammatory lymphocytic foci in the lacrimal gland. Hawley et al. found substantial changes in the myoepithelial cell morphology and contractile protein expression in these relatively normal areas (between the lymphocytic foci) of the lacrimal gland, which is likely due to decreased expression of the contractile proteins of smooth muscle actin and calponin, and significant loss of the oxytocin receptor [49]. Hayashi et al. suggested that acini destruction in lacrimal glands may be induced by systemic and local Th1 cell-dominant reactions [50].

In addition, the corneal nerves play a critical role in the pathophysiology of dry eye because they contribute to the reflex control of basal tear production and blinking [51]. Compared to healthy subjects, both the density and function of sensory nerve fiber terminals in the cornea declined in primary SS [52,53], leading to a vicious circle of corneal hypoesthesia. Moreover, primary SS also results in damage to secretomotor innervation and inflammatory destruction of acini and ducts [54]. SS dry eye is generally described as aqueous-deficient dry eye and much attention has been paid to the lack of tear production [55,56]. However, as a result of the meibomian glands being under immune attack, meibomian glands dysfunction (MGD) is captured in primary SS patients [57], with a diffuse abnormality of the meibomian glands, commonly characterized by chronic terminal duct obstructions and qualitative or quantitative changes in the glandular secretion [58]. Lymphocytic infiltration was also found in the conjunctiva of patients with SS related dry eye [59]. By using a Sjogren’s syndrome model MRL/MpJ-Fas(lpr) mouse, Diebold et al. found lymphocytic infiltration and goblet cell marker alteration in the conjunctiva [60]. Furthermore, desiccating stress caused by SS promotes the maturation of monocytes to antigen-presenting cells with elevated levels of inflammatory genes in conjunctiva [61]. Keratinization of the conjunctival and corneal epithelia, squamous metaplasia, and goblet cell loss are observed in severe primary SS.

In conclusion, SS dry eye is a multifactorial disease, comprising aqueous, lipid, and mucin components, which is comprised of a series of disorders of the lacrimal gland, conjunctiva, corneal and meibomian glands, causing aqueous-deficient or evaporative dry eye.

#### 3.2.2. Rheumatoid Arthritis & Dry Eye

RA is a common systemic inflammatory disease, characterized by persistent synovitis, systemic inflammation, and autoantibodies (particularly rheumatoid factor and citrullinated peptide) [62]. The morbidity ranges from 0.5% to 2% in the overall population [63]. RA affects the joints, but the systemic inflammatory process could affect other tissues and organs, for example, the eyes. Most relevant studies showed that dry eye was the most frequent ocular complication in RA patients, while others include scleritis, episcleritis, keratitis, etc. [64,65,66]. Although some patients with RA can develop secondary SS with the prevalence varying from 4% to 50% [67], dry eye is common even in RA patients without secondary SS [10]. No matter what kind of RA (RA-SS or RA-nonSS), dry eye is the most common ocular manifestation. As for gender characteristics of dry eye in RA, it is more common in women, with a female-to-male ratio of 9:1 [68]. The probability of ocular involvement increases with the duration of the disease and it may represent the main clinical manifestation of RA in patients with long-standing disease. In addition, sometimes ocular complaints are the first symptom of RA patients [64]. The severity of eye dryness was verified to be highly correlated with juvenile RA [69]. However, there was no significant correlation between the severity of dry eye and RA activity. Therefore, the deterioration of the systemic condition of RA does not one hundred percent lead to the aggravation of dry eye symptoms, and dry eye cannot be excluded even in patients with mild RA [10].

Mechanisms of RA are the repeated activation of innate immunity, especially at mucosal surfaces. Innate immunity could activate fibroblast-like synoviocytes, dendritic cells, and macrophages during the earliest phases in individuals with underlying immune hyperreactivity. The overproduction of pro-inflammatory cytokines, such as tumor necrosis factor (TNF) and interleukin-6 (IL-6), lead to the proliferation of synovial cells in joints and subsequent pannus formation, cartilage destruction, and bone erosions [62]. Among ARDs other than SS, the association is the closest between dry eye and RA [70]. As mentioned above, increased deterioration of RA seems not to lead to the aggravation of dry eye. Consequently, the etiology of dry eye is different in SS and non-SS patients with RA [10].

The mechanism of dry eye in RA patients is different from SS and might be a result of local pathology affecting the tear fluid, conjunctiva, or cornea. The pathological changes of the ocular surface in RA-SS are dissimilar to RA-nonSS as discovered by Villani et al. after they detected the concentration of human IL-1a, IL-6, IL-8, and TNF-a (substantial in the synovial fluid and tissue of RA patients) in tear samples [66]. After intervention therapy using blockers against TNF-a, IL-1a, and IL-6, there were remarkable therapeutic efficacies in RA. Additionally, the concentration of IL-1a and IL-6 in the tear ducts of RA-SS patients declined significantly, while there were no evident changes of those cytokines in RA-nonSS patients [66]. However, Usuba et al. demonstrated the evaporative component disturbance in RA patients, with the presence of meibomian gland dysfunction (associated with alterations in the lipid layer of tear film) as well as decreased goblet cell numbers (associated with the mucin layer of the tear film), in addition to the traditional aqueous tear deficiency related to dry eye disease. Furthermore, treatment with TNF inhibitors improved conjunctival goblet cell numbers in RA patients with mild dry eye, which is conducive to relieving dry eye by increasing the secretion of mucin [71]. This is consistent with Tong’s findings that TNF-inhibitor treatment controlled inflammatory disease activity concomitantly to conjunctival cytology, especially the goblet cell density, implying a common underlying inflammatory mechanism for ocular and articular activity [72]. In addition, in a clinical trial by Schargus et al., tear film hyperosmolarity highly correlates (>316 mOsmol/L) with undiagnosed dry eye in RA patients [73].

Most of the current studies demonstrated that dry eye in RA tends to correlate with local pathogenic mechanisms other than with systemic inflammation [10,73]. In summary, mixing aqueous-deficient dry eye and evaporative dry eye, RA-nonSS dry eye comprises disorders of the conjunctiva and meibomian gland, resulting from a decrease of aqueous, lipid, and mucin components. Findings of note include that the meibomian gland dysfunction is associated with higher disease activity parameters, and that TNF-inhibitor therapy improves ocular surface health by promoting the recovery of goblet cells.

#### 3.2.3. Systemic Lupus Erythematosus & Dry Eye

SLE is a multisystem, chronic, autoimmune disease that mostly affects women (female/male ratio ranging from 6:1 to 10:1) who are of child-bearing age [74]. Ocular symptoms can be detected in approximately one-third of SLE patients. The incidence of SS secondary to SLE is about 13.96% [75]. Dry eye complaints are usually ignored in SLE patients without secondary SS. However, dry eye is the most common ocular manifestation of SLE [11], and the same is true of juvenile systemic lupus erythematosus [76]. As a significant “window” into SLE, dry eye is diagnosed with a prevalence of 5% in early-onset SLE patients, 16% in the duration stage, and 33% in late-onset [77].

Elevated levels of anti-double-stranded DNA antibodies (anti-dsDNA) and erythrocyte sedimentation rate [78], as well as the depression in complement levels (C3 and C4) [79,80] are commonly used to predict lupus’s flare and assess its activity. Chen et al. found that in SLE patients without secondary SS, the progress of dry eye severity was consistent with anti-dsDNA titers and low C3 levels, but not with the low C4 levels, erythrocyte sedimentation rate, and antinuclear antibodies (ANAs) [81]. SLE alters not only the density and the morphology of the corneal Langerhans cells, but it also interferes with corneal homeostasis and might contribute to the development of dry eye [82].

As significant risk factors for dry eye, poorer meibomian gland function and worse tear film lipid layers were also found in SLE patients without secondary SS, leading to decreased tear volume and faster evaporation [83]. SLE tends to present with multiple factors of induced dry eye, including disorders of the lacrimal gland, cornea, and meibomian glands.

#### 3.2.4. Systemic Sclerosis & Dry Eye

Scleroderma, also called SSc, is an immune-mediated rheumatic disease that is characterized by fibrosis and vasculopathy of the skin and internal organs [84]. The proportion of females with SSc is higher than that of males (female/male = 3:1) [85]. This disease involves different organs, including the eyes and periocular tissues. Dry eye is the most common ocular manifestation of SSc.

SSc related dry eye is likely owing to the fibrosis of the conjunctiva and lacrimal glands [86]. Based on a histological study, the fibrosis of the conjunctiva is associated with degranulating mast cells. Dry eye from primary SS and dry eye from SSc share a similar mechanism since the primary lacrimal gland duct is involved in both. However, the lymphoid infiltration in the gland is sparser in SSc [15], which can be used to differentiate SSc and primary SS related dry eye. The presence of fibrosis suggests SSc, whereas lymphocytic infiltration is a critical sign of primary SS [59]. Because SSc is related to peripheral neuropathy, de A. F. Gomes et al. held the opinion that patients with SSc may have a decreased corneal sensation, which can explain the lack of correlation between signs and symptoms of dry eye [15].

In addition to the impairment of the aqueous layer of the tear film due to lacrimal gland fibrosing [86], blepharitis and MGD are other factors for SSc related dry eye. The lacrimal gland, conjunctiva, cornea, and meibomian glands are all involved in SSc related dry eye. Characteristics of primary SS, RA, SLE, and SSc related dry eye disease are shown in Table 1.

## 4. ARDs Related Dry Eye and Its Association with Retinopathy

### 4.1. How Dry Eye Disease Affects Retinal Diseases

Dry eye is related to some retinal diseases directly or indirectly. Systemic diseases or exposure to hazardous substances should be considered if the dry eye and retinal diseases are concurrent.

Dry eye and retinal diseases both belong to the catalog of age-related ocular diseases, including age-related macular degeneration, cataracts, and glaucoma. These above-mentioned changes may be attributed to similar pathophysiological mechanisms, including dysregulated mitochondrial metabolism, reprogrammed glucose metabolism, and impaired methylation in the aging eye [87]. The overlap of endocrine (hormonal withdrawal) and neuronal dysfunction with aging contribute to age-related ocular diseases [88]. The age-related ocular diseases can be improved in various parameters of visual function by using L-carnitine and its derivatives [89].

The environment is one of the most significant risks for dry eye. Endocrine disrupting chemicals (EDCs) are a group of chemical compounds that can interfere with endocrine hormone homeostasis, including about 1000 synthesized chemicals. Humans are exposed to EDCs from their diet, thermal receipts, personal care products, antimicrobial soaps, household or agricultural pesticides, and cleaning products, leading to physiological abnormalities in the body, such as ocular surface disorders, including dry eye [90]. The induction of chronic physiological abnormalities caused by EDCs may subsequently result in retina diseases, including age-related macular degeneration, retinitis, retinal detachment, and diabetic retinopathy [91].

In addition, the ocular surface and retina are often involved in diabetes. Diabetes is associated with progressive damage to corneal nerves and epithelial cells, which can increase the risk of dry eye [92]. Up to 54% of type 2 diabetic patients suffer from dry eye syndrome, which is more frequent in diabetic patients with diabetic retinopathy [93].

Alpha-lipoic acid showed similar therapeutic effects in the treatment of both dry eye disease and diabetic retinopathy [94].

### 4.2. How ARDs Affect Retinal Diseases

The retina is often involved in ARDs and commonly associated with systemic inflammatory cell infiltration, which may constitute the initial presentation of systemic immune-mediated disorders. Retinal vasculitis may also represent the most common retinal manifestations of the rheumatic disease and it is often accompanied by uveitis, scleritis, or macular edema [95]. It was reported that about 51% of SSc patients had different forms of retinal diseases, including mild retinal pigment epithelial atrophy, drusen, choroidal scar formation, and severe age-related macular degeneration [96]. Retinal involvement, the second most common ocular manifestation among SLE patients, has a prevalence of 7–29% and is one of the most common indicators of a highly-active period of SLE [97].

Since a case of optic neuropathy secondary to SS was first reported in 1990, more and more studies are focusing on the relationship between SS and retinopathy [98]. With the continuous improvement of retinal detecting technology, more details of the mechanisms have been uncovered by recent studies. According to a clinical study of patients with SS, anti-SSB autoantibodies might be a useful marker to predict abnormally reduced peripapillary retinal nerve fiber layers and macular ganglion cell–inner plexiform layer thickness [99]. Furthermore, as reported recently, optical coherence tomography angiography was used to investigate the differences in retinal thickness and superficial vascular density between patients with SS and healthy controls, suggesting the retinal thinning of the macular area can also reflect the severity of dry eye in SS and has clinical value for assisted imaging diagnosis [100]. Similarly, in RA, retinal capillary plexus density in the macula is lower than in normal individuals [101]. In addition, even if no clinical and ophthalmoscopic signs are present, retinal vessel inflammation is present in 18% of RA patients and can represent one of the possible extra-articular manifestations of RA [102].

## 5. Clinical Attention–Dry Eye Is the “Window” of ARDs

First, dry eye is a challenge for patients and may be difficult for clinicians to diagnose and manage effectively [103]. Moreover, patients ultimately diagnosed with an autoimmune rheumatic disease are also a diagnostic challenge [6]. We should devote more attention to symptoms of ARD related dry eye. Second, there is a sequence of other ocular disorders that ARDs patients may have, e.g., scleritis, episcleritis, keratitis, etc. However, the symptoms and signs of these ocular diseases are easy to notice, unlike the latent dry eye. Third, the eye could be a surrogate marker for the onset or aggravation of an immune reactivation in ARDs since dry eye complaints can antedate the diagnosis of rheumatic diseases. It is possible to avoid the delay of many long-term sequelae by recognizing dry eye manifestations of ARDs.

Of note, a significant percentage of dry eye diagnoses can be missed because of the number of symptoms just for diagnosis of dry eye as well as isolated inclusion criteria. Severe dry eye is usually common in patients with ARDs. In addition, the decreased corneal sensation is a feature of severe dry eye which might alter the patient’s perception of symptoms of ocular irritation. The reduced corneal sensation could provide inadequate feedback through the ophthalmic nerve to the central nervous system, resulting in less efferent stimulation to the lacrimal gland, reduced tear production, and the promotion of a vicious cycle [104]. Overall, examines related to dry eye are significant and should be implemented in patients with ARDs, even if there is no specific ocular complaint.

## 6. Conclusions

Dry eye is the most common ocular complication in ARDs. ARD related dry eye, caused by functional disorders of the lacrimal gland, lacrimal duct, conjunctiva, cornea, and meibomian glands, includes the two kinds of aqueous-deficient and evaporative dry eye, simultaneously. Severe symptoms of dry eye are usually presented in patients with ARDs, which might enable the dry eye to be a significant indicator of ARDs. In the future, the research on ARD related dry eye might contribute to the investigation of systemically immunological and molecular mechanisms in ARDs. Significantly, because of the direct or indirect associations of ARD related dry eye with some retinal diseases, a comprehensive understanding of dry eye, ARDs, and relative complications is necessary.

## Figures and Tables

**Table 1:** Characteristics of autoimmune rheumatic diseases related dry eye disease.

	Primary Sjogren’s Syndrome	Rheumatoid Arthritis	Systemic Lupus Erythematosus	Systemic Sclerosis
Incidence of Dry Eye	95%	38–47%	13.4–39.5%	37–79%
Incidence of Secondary Sjogren’s Syndrome	-	4–50%	13.96%	14–60%
The Most Common Ocular Manifestation	dry eye	dry eye	dry eye	dry eye
Correlation between Dry Eye and ARDs Activity	positive relationship	uncertain in adults, a positive relationship between juvenile RA and dry eye	positive relationship	positive relationship
Ocular Structures Involved	♦ lacrimal gland ♦ acini and ducts of lacrimal gland ♦ conjunctiva ♦ corneal ♦ meibomian gland	♦ conjunctiva ♦ cornea ♦ meibomian gland	♦ lacrimal gland ♦ cornea ♦ meibomian gland	♦ lacrimal gland ♦ conjunctiva ♦ cornea ♦ especially meibomian gland
Type of Dry Eye	aqueous-deficient and evaporative dry eye	aqueous-deficient and evaporative dry eye	aqueous-deficient and evaporative dry eye	aqueous-deficient and mainly evaporative dry eye
Changes in Tear Film	aqueous, lipid, and mucin	aqueous, lipid, and mucin	aqueous, lipid, and mucin	aqueous, mucin especially lipid

Note: ARDs, autoimmune rheumatic diseases.

## Data Availability

Not applicable.

## References

[1] Craig J.P., Nichols K.K., Akpek E.K., Caffery B., Dua H.S., Joo C.K., Liu Z., Nelson J.D., Nichols J.J., Tsubota K. (2017). TFOS DEWS II Definition and Classification Report. Ocul. Surf..

[2] Clayton J.A. (2018). Dry Eye. N. Engl. J. Med..

[3] Stapleton F., Alves M., Bunya V.Y., Jalbert I., Lekhanont K., Malet F., Na K.S., Schaumberg D., Uchino M., Vehof J. (2017). TFOS DEWS II Epidemiology Report. Ocul. Surf..

[4] Mehra D., Galor A. (2020). Digital Screen Use and Dry Eye: A Review. Asia Pac. J. Ophthalmol..

[5] Mandell J.T., Idarraga M., Kumar N., Galor A. (2020). Impact of Air Pollution and Weather on Dry Eye. J. Clin. Med..

[6] Goldblatt F., O′Neill S.G. (2013). Clinical aspects of autoimmune rheumatic diseases. Lancet.

[7] Periman L.M., Perez V.L., Saban D.R., Lin M.C., Neri P. (2020). The Immunological Basis of Dry Eye Disease and Current Topical Treatment Options. J. Ocul. Pharmacol. Ther..

[8] Akpek E.K., Amescua G., Farid M., Garcia-Ferrer F.J., Lin A., Rhee M.K., Varu D.M., Musch D.C., Dunn S.P., Mah F.S. (2019). Dry Eye Syndrome Preferred Practice Pattern^®^. Ophthalmology.

[9] Kemeny-Beke A., Szodoray P. (2020). Ocular manifestations of rheumatic diseases. Int. Ophthalmol..

[10] Fujita M., Igarashi T., Kurai T., Sakane M., Yoshino S., Takahashi H. (2005). Correlation between dry eye and rheumatoid arthritis activity. Am. J. Ophthalmol..

[11] Dammacco R. (2018). Systemic lupus erythematosus and ocular involvement: An overview. Clin. Exp. Med..

[12] El-Shereef R.R., Mohamed A.S., Hamdy L. (2013). Ocular manifestation of systemic lupus erythematosus. Rheumatol. Int..

[13] Mavragani C.P., Moutsopoulos H.M. (2014). Sjögren syndrome. CMAJ.

[14] Ramos-Casals M., Brito-Zeron P., Siso-Almirall A., Bosch X. (2012). Primary Sjogren syndrome. BMJ.

[15] de AF Gomes B., Santhiago M.R., de Azevedo M.N., Moraes H.V. (2012). Evaluation of dry eye signs and symptoms in patients with systemic sclerosis. Graefes Arch. Clin. Exp. Ophthalmol..

[16] Helmick C.G., Felson D.T., Lawrence R.C., Gabriel S., Hirsch R., Kwoh C.K., Liang M.H., Kremers H.M., Mayes M.D., Merkel P.A. (2008). Estimates of the prevalence of arthritis and other rheumatic conditions in the United States. Part I. Arthritis Rheum..

[17] Yu K., Bunya V., Maguire M., Asbell P., Ying G.-S. (2021). Systemic Conditions Associated with Severity of Dry Eye Signs and Symptoms in the Dry Eye Assessment and Management Study. Ophthalmology.

[18] Paulsen A.J., Cruickshanks K.J., Fischer M.E., Huang G.-H., Klein B.E.K., Klein R., Dalton D.S. (2014). Dry eye in the beaver dam offspring study: Prevalence, risk factors, and health-related quality of life. Am. J. Ophthalmol..

[19] Rosas J., Sánchez-Piedra C., Fernández-Castro M., Andreu J.L., Martínez-Taboada V., Olivé A. (2019). ESSDAI activity index of the SJÖGRENSER cohort: Analysis and comparison with other European cohorts. Rheumatol. Int..

[20] Abd-Allah N.M., Hassan A.A., Omar G., Hamdy M., Abdelaziz S.T.A., Abd El Hamid W.M., Moussa R.A. (2020). Dry eye in rheumatoid arthritis: Relation to disease activity. Immunol. Med..

[21] Arnaud L., Mathian A., Boddaert J., Amoura Z. (2012). Late-onset systemic lupus erythematosus: Epidemiology, diagnosis and treatment. Drugs Aging.

[22] Pflugfelder S.C., de Paiva C.S. (2017). The Pathophysiology of Dry Eye Disease: What We Know and Future Directions for Research. Ophthalmology.

[23] De Paiva C.S., Chotikavanich S., Pangelinan S.B., Pitcher J.D., Fang B., Zheng X., Ma P., Farley W.J., Siemasko K.F., Niederkorn J.Y. (2009). IL-17 disrupts corneal barrier following desiccating stress. Mucosal. Immunol..

[24] García-Posadas L., Hodges R.R., Li D., Shatos M.A., Storr-Paulsen T., Diebold Y., Dartt D.A. (2016). Interaction of IFN-γ with cholinergic agonists to modulate rat and human goblet cell function. Mucosal. Immunol..

[25] Bron A.J., de Paiva C.S., Chauhan S.K., Bonini S., Gabison E.E., Jain S., Knop E., Markoulli M., Ogawa Y., Perez V. (2017). TFOS DEWS II pathophysiology report. Ocul. Surf..

[26] Wieczorek R., Jakobiec F.A., Sacks E.H., Knowles D.M. (1988). The immunoarchitecture of the normal human lacrimal gland. Relevancy for understanding pathologic conditions. Ophthalmology.

[27] Bozic B., Pruijn G.J., Rozman B., van Venrooij W.J. (1993). Sera from patients with rheumatic diseases recognize different epitope regions on the 52-kD Ro/SS-A protein. Clin. Exp. Immunol..

[28] Patel S., Hwang J., Mehra D., Galor A. (2021). Corneal Nerve Abnormalities in Ocular and Systemic Diseases. Exp. Eye Res..

[29] Villani E., Galimberti D., Viola F., Mapelli C., Ratiglia R. (2007). The cornea in Sjogren’s syndrome: An in vivo confocal study. Investig. Ophthalmol. Vis. Sci..

[30] Benítez del Castillo J.M., Wasfy M.A.S., Fernandez C., Garcia-Sanchez J. (2004). An in vivo confocal masked study on corneal epithelium and subbasal nerves in patients with dry eye. Investig. Ophthalmol. Vis. Sci..

[31] Xu K.P., Yagi Y., Tsubota K. (1996). Decrease in corneal sensitivity and change in tear function in dry eye. Cornea.

[32] Ogawa Y. (2018). Sjögren’s Syndrome, Non-Sjögren’s Syndrome, and Graft-Versus-Host Disease Related Dry Eye. Investig. Ophthalmol. Vis. Sci..

[33] Moon J., Yoon C.H., Choi S.H., Kim M.K. (2020). Can Gut Microbiota Affect Dry Eye Syndrome?. Int. J. Mol. Sci..

[34] Kowalski E.N., Qian G., Vanni K.M.M., Sparks J.A. (2022). A Roadmap for Investigating Preclinical Autoimmunity Using Patient-Oriented and Epidemiologic Study Designs: Example of Rheumatoid Arthritis. Front. Immunol..

[35] Wang Y., Chen S., Chen J., Xie X., Gao S., Zhang C., Zhou S., Wang J., Mai R., Lin Q. (2020). Germline genetic patterns underlying familial rheumatoid arthritis, systemic lupus erythematosus and primary Sjögren’s syndrome highlight T cell-initiated autoimmunity. Ann. Rheum. Dis..

[36] de Paiva C.S., Trujillo-Vargas C.M., Schaefer L., Yu Z., Britton R.A., Pflugfelder S.C. (2021). Differentially Expressed Gene Pathways in the Conjunctiva of Sjögren Syndrome Keratoconjunctivitis Sicca. Front. Immunol..

[37] Mavragani C.P., Moutsopoulos H.M. (2010). The geoepidemiology of Sjogren’s syndrome. Autoimmun. Rev..

[38] Haugen A.J., Peen E., Hulten B., Johannessen A.C., Brun J.G., Halse A.K., Haga H.J. (2008). Estimation of the prevalence of primary Sjogren’s syndrome in two age-different community-based populations using two sets of classification criteria: The Hordaland Health Study. Scand. J. Rheumatol..

[39] Gøransson L.G., Haldorsen K., Brun J.G., Harboe E., Jonsson M.V., Skarstein K., Time K., Omdal R. (2011). The point prevalence of clinically relevant primary Sjögren’s syndrome in two Norwegian counties. Scand. J. Rheumatol..

[40] Jonsson R., Vogelsang P., Volchenkov R., Espinosa A., Wahren-Herlenius M., Appel S. (2011). The complexity of Sjogren’s syndrome: Novel aspects on pathogenesis. Immunol. Lett..

[41] Caffery B., Simpson T., Wang S., Bailey D., McComb J., Rutka J., Slomovic A., Bookman A. (2010). Rose bengal staining of the temporal conjunctiva differentiates Sjogren’s syndrome from keratoconjunctivitis sicca. Investig. Ophthalmol. Vis. Sci..

[42] Ronnblom L. (2011). The type I interferon system in the etiopathogenesis of autoimmune diseases. Upsala J. Med. Sci..

[43] Fayyaz A., Kurien B.T., Scofield R.H. (2016). Autoantibodies in Sjogren’s Syndrome. Rheum. Dis. Clin. N. Am..

[44] Vosters J.L., Landek-Salgado M.A., Yin H., Swaim W.D., Kimura H., Tak P.P., Caturegli P., Chiorini J.A. (2009). Interleukin-12 induces salivary gland dysfunction in transgenic mice, providing a new model of Sjogren’s syndrome. Arthritis Rheum..

[45] Turpie B., Yoshimura T., Gulati A., Rios J.D., Dartt D.A., Masli S. (2009). Sjögren’s Syndrome-Like Ocular Surface Disease in Thrombospondin-1 Deficient Mice. Am. J. Pathol..

[46] De Paiva C.S., Hwang C.S., Pitcher J.D., Pangelinan S.B., Rahimy E., Chen W., Yoon K.C., Farley W.J., Niederkorn J.Y., Stern M.E. (2010). Age-related T-cell cytokine profile parallels corneal disease severity in Sjogren’s syndrome-like keratoconjunctivitis sicca in CD25KO mice. Rheumatology.

[47] Kosenda K., Ichii O., Otsuka S., Hashimoto Y., Kon Y. (2013). BXSB/MpJ-Yaa mice develop autoimmune dacryoadenitis with the appearance of inflammatory cell marker messenger RNAs in the lacrimal fluid. Clin. Exp. Ophthalmol..

[48] Hayashi T., Hayashi H., Fujii T., Adachi C., Hasegawa K. (2008). Ultrastructure of myoepithelial cells as a target cell in sialoadenitis of submandibular glands of lupus-prone female NZBxNZWF1 mice. Virchows Arch..

[49] Hawley D., Tang X., Zyrianova T., Shah M., Janga S., Letourneau A., Schicht M., Paulsen F., Hamm-Alvarez S., Makarenkova H.P. (2018). Myoepithelial cell-driven acini contraction in response to oxytocin receptor stimulation is impaired in lacrimal glands of Sjögren’s syndrome animal models. Sci. Rep..

[50] Hayashi T., Shimoyama N., Mizuno T. (2012). Destruction of salivary and lacrimal glands by Th1-polarized reaction in a model of secondary Sjogren’s syndrome in lupus-prone female NZB x NZWF(1) mice. Inflammation.

[51] Belmonte C., Nichols J.J., Cox S.M., Brock J.A., Begley C.G., Bereiter D.A., Dartt D.A., Galor A., Hamrah P., Ivanusic J.J. (2017). TFOS DEWS II pain and sensation report. Ocul. Surf..

[52] Villani E., Magnani F., Viola F., Santaniello A., Scorza R., Nucci P., Ratiglia R. (2013). In vivo confocal evaluation of the ocular surface morpho-functional unit in dry eye. Optom. Vis. Sci..

[53] Li F., Zhang Q., Ying X., He J., Jin Y., Xu H., Cheng Y., Zhao M. (2021). Corneal nerve structure in patients with primary Sjogren’s syndrome in China. BMC Ophthalmol..

[54] Zeng M., Szymczak M., Ahuja M., Zheng C., Yin H., Swaim W., Chiorini J.A., Bridges R.J., Muallem S. (2017). Restoration of CFTR Activity in Ducts Rescues Acinar Cell Function and Reduces Inflammation in Pancreatic and Salivary Glands of Mice. Gastroenterology.

[55] Akpek E.K., Bunya V.Y., Saldanha I.J. (2019). Sjögren’s Syndrome: More Than Just Dry Eye. Cornea.

[56] Liew M.S., Zhang M., Kim E., Akpek E.K. (2012). Prevalence and predictors of Sjogren’s syndrome in a prospective cohort of patients with aqueous-deficient dry eye. Br. J. Ophthalmol..

[57] Zi C., Huang Q., Ren Y., Yao H., He T., Gao Y. (2021). Meibomian gland dysfunction and primary Sjogren’s syndrome dry eye: A protocol for systematic review and meta-analysis. BMJ Open.

[58] Menzies K.L., Srinivasan S., Prokopich C.L., Jones L. (2015). Infrared imaging of meibomian glands and evaluation of the lipid layer in Sjogren’s syndrome patients and nondry eye controls. Investig. Ophthalmol. Vis. Sci..

[59] Mancel E., Janin A., Gosset D., Hatron P.Y., Gosselin B. (1993). Conjunctival Biopsy in Scleroderma and Primary Sjögren’s Syndrome. Am. J. Ophthalmol..

[60] Diebold Y., Chen L.-L., Tepavcevic V., Ferdman D., Hodges R.R., Dartt D.A. (2007). Lymphocytic infiltration and goblet cell marker alteration in the conjunctiva of the MRL/MpJ-Fas(lpr) mouse model of Sjögren’s syndrome. Exp. Eye Res..

[61] Alam J., de Paiva C.S., Pflugfelder S.C. (2021). Desiccation Induced Conjunctival Monocyte Recruitment and Activation-Implications for Keratoconjunctivitis. Front. Immunol..

[62] Scott D.L., Wolfe F., Huizinga T.W.J. (2010). Rheumatoid arthritis. Lancet.

[63] Minichiello E., Semerano L., Boissier M.C. (2016). Time trends in the incidence, prevalence, and severity of rheumatoid arthritis: A systematic literature review. Jt. Bone Spine.

[64] Conforti A., Di Cola I., Pavlych V., Ruscitti P., Berardicurti O., Ursini F., Giacomelli R., Cipriani P. (2021). Beyond the joints, the extra-articular manifestations in rheumatoid arthritis. Autoimmun. Rev..

[65] Murray P.I., Rauz S. (2016). The eye and inflammatory rheumatic diseases: The eye and rheumatoid arthritis, ankylosing spondylitis, psoriatic arthritis. Best Pract. Res. Clin. Rheumatol..

[66] Villani E., Galimberti D., Del Papa N., Nucci P., Ratiglia R. (2013). Inflammation in dry eye associated with rheumatoid arthritis: Cytokine and in vivo confocal microscopy study. Innate Immun..

[67] Theander E., Jacobsson L.T. (2008). Relationship of Sjogren’s syndrome to other connective tissue and autoimmune disorders. Rheum. Dis. Clin. N. Am..

[68] Choudhary M.M., Hajj-Ali R.A., Lowder C.Y. (2014). Gender and ocular manifestations of connective tissue diseases and systemic vasculitides. J. Ophthalmol..

[69] El-Shazly A.A., Mohamed A.A. (2012). Relation of dry eye to disease activity in juvenile rheumatoid arthritis. Eur. J. Ophthalmol..

[70] Lemp M.A. (2005). Dry eye (Keratoconjunctivitis Sicca), rheumatoid arthritis, and Sjogren’s syndrome. Am. J. Ophthalmol..

[71] Usuba F.S., de Medeiros-Ribeiro A.C., Novaes P., Aikawa N.E., Bonfiglioli K., Santo R.M., Bonfa E., Alves M.R. (2020). Dry eye in rheumatoid arthritis patients under TNF-inhibitors: Conjunctival goblet cell as an early ocular biomarker. Sci. Rep..

[72] Tong L., Thumboo J., Tan Y.K., Wong T.Y., Albani S. (2014). The eye: A window of opportunity in rheumatoid arthritis?. Nat. Rev. Rheumatol..

[73] Schargus M., Wolf F., Tony H.-P., Meyer-Ter-Vehn T., Geerling G. (2014). Correlation between tear film osmolarity, dry eye disease, and rheumatoid arthritis. Cornea.

[74] Somers E.C., Marder W., Cagnoli P., Lewis E.E., DeGuire P., Gordon C., Helmick C.G., Wang L., Wing J.J., Dhar J.P. (2014). Population-based incidence and prevalence of systemic lupus erythematosus: The Michigan Lupus Epidemiology and Surveillance program. Arthritis Rheumatol..

[75] Alani H., Henty J.R., Thompson N.L., Jury E., Ciurtin C. (2018). Systematic review and meta-analysis of the epidemiology of polyautoimmunity in Sjogren’s syndrome (secondary Sjogren’s syndrome) focusing on autoimmune rheumatic diseases. Scand. J. Rheumatol..

[76] Gawdat G., El-Fayoumi D., Marzouk H., Farag Y. (2018). Ocular Manifestations in Children with Juvenile-Onset Systemic Lupus Erythematosus. Semin. Ophthalmol..

[77] Cervera R., Khamashta M.A., Font J., Sebastiani G.D., Gil A., Lavilla P., Doménech I., Aydintug A.O., Jedryka-Góral A., de Ramón E. (1993). Systemic lupus erythematosus: Clinical and immunologic patterns of disease expression in a cohort of 1,000 patients. The European Working Party on Systemic Lupus Erythematosus. Medicine.

[78] Farland L.V., Missmer S.A., Bijon A., Gusto G., Gelot A., Clavel-Chapelon F., Mesrine S., Boutron-Ruault M.C., Kvaskoff M. (2017). Associations among body size across the life course, adult height and endometriosis. Hum. Reprod..

[79] Mirzayan M.J., Schmidt R.E., Witte T. (2000). Prognostic parameters for flare in systemic lupus erythematosus. Rheumatology.

[80] Smith P.P., Gordon C. (2010). Systemic lupus erythematosus: Clinical presentations. Autoimmun. Rev..

[81] Chen A., Chen H.T., Hwang Y.H., Chen Y.T., Hsiao C.H., Chen H.C. (2016). Severity of dry eye syndrome is related to anti-dsDNA autoantibody in systemic lupus erythematosus patients without secondary Sjogren syndrome: A cross-sectional analysis. Medicine.

[82] Resch M.D., Marsovszky L., Nemeth J., Bocskai M., Kovacs L., Balog A. (2015). Dry eye and corneal langerhans cells in systemic lupus erythematosus. J. Ophthalmol..

[83] Gu Z., Lu Q., Zhang A., Shuai Z.W., Liao R. (2022). Analysis of Ocular Surface Characteristics and Incidence of Dry Eye Disease in Systemic Lupus Erythematosus Patients Without Secondary Sjogren’s Syndrome. Front. Med..

[84] Denton C.P., Khanna D. (2017). Systemic sclerosis. Lancet.

[85] Chifflot H., Fautrel B., Sordet C., Chatelus E., Sibilia J. (2008). Incidence and prevalence of systemic sclerosis: A systematic literature review. Semin. Arthritis Rheum..

[86] Tailor R., Gupta A., Herrick A., Kwartz J. (2009). Ocular manifestations of scleroderma. Surv. Ophthalmol..

[87] Wang Y., Grenell A., Zhong F., Yam M., Hauer A., Gregor E., Zhu S., Lohner D., Zhu J., Du J. (2018). Metabolic signature of the aging eye in mice. Neurobiol. Aging.

[88] Cascio C., Deidda I., Russo D., Guarneri P. (2015). The estrogenic retina: The potential contribution to healthy aging and age-related neurodegenerative diseases of the retina. Steroids.

[89] Pescosolido N., Imperatrice B., Karavitis P. (2008). The aging eye and the role of L-carnitine and its derivatives. Drugs R D.

[90] Pontelli R.C.N., Rocha B.A., Garcia D.M., Pereira L.A., Souza M.C.O., Barbosa F., Rocha E.M. (2020). Endocrine disrupting chemicals associated with dry eye syndrome. Ocul. Surf..

[91] Li M., Yang T., Gao L., Xu H. (2021). An inadvertent issue of human retina exposure to endocrine disrupting chemicals: A safety assessment. Chemosphere.

[92] Han S.B., Yang H.K., Hyon J.Y. (2019). Influence of diabetes mellitus on anterior segment of the eye. Clin. Interv. Aging.

[93] Manaviat M.R., Rashidi M., Afkhami-Ardekani M., Shoja M.R. (2008). Prevalence of dry eye syndrome and diabetic retinopathy in type 2 diabetic patients. BMC Ophthalmol..

[94] Ajith T.A. (2020). Alpha-lipoic acid: A possible pharmacological agent for treating dry eye disease and retinopathy in diabetes. Clin. Exp. Pharmacol. Physiol..

[95] Androudi S., Dastiridou A., Symeonidis C., Kump L., Praidou A., Brazitikos P., Kurup S.K. (2013). Retinal vasculitis in rheumatic diseases: An unseen burden. Clin. Rheumatol..

[96] Szucs G., Szekanecz Z., Aszalos Z., Gesztelyi R., Zsuga J., Szodoray P., Kemeny-Beke A. (2021). A Wide Spectrum of Ocular Manifestations Signify Patients with Systemic Sclerosis. Ocul. Immunol. Inflamm..

[97] Conigliaro P., Cesareo M., Chimenti M.S., Triggianese P., Canofari C., Barbato C., Giannini C., Salandri A.G., Nucci C., Perricone R. (2019). Take a look at the eyes in Systemic Lupus Erythematosus: A novel point of view. Autoimmun. Rev..

[98] Berman J.L., Kashii S., Trachtman M.S., Burde R.M. (1990). Optic neuropathy and central nervous system disease secondary to Sjögren’s syndrome in a child. Ophthalmology.

[99] Yang J.M., Heo H., Park S.W. (2014). Relationship between retinal morphological findings and autoantibody profile in primary Sjögren’s syndrome. Jpn. J. Ophthalmol..

[100] Liu R., Wang Y., Li Q., Xia Q., Xu T., Han T., Cai S., Luo S., Wu R., Shao Y. (2022). Optical Coherence Tomography Angiography Biomarkers of Retinal Thickness and Microvascular Alterations in Sjogren’s Syndrome. Front. Neurol..

[101] Ayar K., Can M.E., Koca N., Çelik D.Ş. (2021). Evaluation of retinal vascularization by optical coherence tomography angiography (OCTA) in rheumatoid arthritis, and its relationship with disease activity. Mod. Rheumatol..

[102] Giordano N., D’Ettorre M., Biasi G., Fioravanti A., Moretti L., Marcolongo R. (1990). Retinal vasculitis in rheumatoid arthritis: An angiographic study. Clin. Exp. Rheumatol..

[103] Hakim F.E., Farooq A.V. (2022). Dry Eye Disease: An Update in 2022. JAMA.

[104] Vitale S., Goodman L.A., Reed G.F., Smith J.A. (2004). Comparison of the NEI-VFQ and OSDI questionnaires in patients with Sjogren’s syndrome-related dry eye. Health Qual. Life Outcomes.

